# Osteosarcopenia as a risk factor for fractures and mortality – 19-year follow-up of a population-based sample

**DOI:** 10.1007/s40520-025-03229-8

**Published:** 2025-11-06

**Authors:** Matias Blomqvist, Maria S. Nuotio, Katri Sääksjärvi, Jaana Pentti BSc, Seppo Koskinen, Sari Stenholm

**Affiliations:** 1 Department of Geriatric Medicine, Turku University Hospital and University of Turku, Häppiläntie 30, 20900 Turku, Finland; 2 Welfare Epidemiology and Monitoring Unit, Finnish Institute for Health and Welfare, Helsinki, Finland; 3 Clinicum, Faculty of Medicine, University of Helsinki, Helsinki, Finland; 4 Department of Public Health, University of Turku, Turku University Hospital, Turku, Finland; 5 Centre for Population Health Research, University of Turku and Turku University Hospital, Turku, Finland; 6 Research Services, Turku University Hospital and University of Turku, Turku, Finland

**Keywords:** Osteosarcopenia, Sarcopenia, Osteoporosis, Fracture, Mortality, Risk, Older adults

## Abstract

**Purpose:**

This study assessed osteoporosis, sarcopenia and osteosarcopenia as risk factors for fractures and mortality during 19-year follow-up.

**Methods:**

We analyzed 2506 individuals aged ≥ 55 from the Finnish Health 2000 Survey. Probable sarcopenia was defined as grip strength < 27 kg for men and < 16 kg for women. Osteoporosis was identified by a bone density T-score < -2,5 via ultrasound or a self-reported diagnosis. Participants were categorized in four groups: reference group with no sarcopenia and no osteoporosis, probable sarcopenia only, osteoporosis only, or osteosarcopenia. Fractures and deaths were identified from national registers until 2019. Four adjustment models were used, adjusting for age, sex, smoking, education, physical activity, and mobility limitation.

**Results:**

Over a mean follow-up of 19.1 years, 580 (23.1%) participants sustained a low-energy fracture of any type and 1,375 (54.9%) died. Osteosarcopenia, probable sarcopenia and osteoporosis were all associated with increased risk of any fracture and mortality compared to the reference group. Osteoporosis alone was associated with lower mortality than osteosarcopenia (HR 0.69, 95% CI 0.50–0.95), but mortality did not differ between probable sarcopenia and osteosarcopenia (HR 0.79, 95% CI 0.59–1.06). No differences in the fracture risk between osteosarcopenia, sarcopenia and osteoporosis were observed.

**Conclusion:**

While both sarcopenia and osteoporosis increase fracture and mortality risks, their combination does not seem to additively elevate fracture risks. Osteoporosis is a stronger predictor for future fractures, whereas probable sarcopenia is more closely linked to mortality. Further research is warranted to determine the best ways to incorporate sarcopenia assessment into comprehensive fracture risk evaluation.

**Supplementary Information:**

The online version contains supplementary material available at 10.1007/s40520-025-03229-8.

## Introduction

Osteoporosis is a systemic skeletal disorder characterized by low bone mineral density and microarchitectural deterioration of bone tissue, and has long been established as a significant risk factor for fractures, particularly those of the spine, hip, distal forearm and proximal humerus [1, 2]. Osteoporotic fractures are associated with significant morbidity, reduced quality of life, and increased mortality [1, 2]. 

Sarcopenia, an age-related condition characterized by muscle wasting and weakness, has been linked to an increased risk of any fracture, spine fracture and hip fracture [3–6]. Furthermore, sarcopenia is recognized as a risk factor for mortality [7], with the increased risk likely arising from various pathways, including cardiovascular, respiratory, and neurodegenerative diseases [4]. Although sarcopenia is defined by low muscle strength combined with low muscle mass, research indicates that low muscle strength is more strongly associated with morbidity and mortality than low muscle mass alone [8]. A diagnosis of probable sarcopenia is made when an individual exhibits low grip strength, even in the absence of muscle mass measurements [4]. 

Both osteoporosis and sarcopenia are known to increase the risk of fractures, and sarcopenia is a risk factor for falls [3]. Therefore, it might be expected that their combination, known as osteosarcopenia, would lead to an even greater fracture risk than either condition alone. Osteosarcopenia is considered a geriatric syndrome that can help identify patients at high risk for fractures and related health issues [9]. A recent meta-analysis suggests that osteosarcopenia increases the risk of falls, fractures, and mortality [10]. This meta-analysis, however, does not give insight into whether osteosarcopenia is a stronger risk factor for these adverse events than sarcopenia or osteoporosis alone.

To the best of our knowledge, only two prospective studies have examined the combined effect of low grip strength or low muscle mass, and low bone mineral density (BMD) on risk of fracture and mortality. Neither of these studies found evidence that osteosarcopenia poses a greater risk for fractures or mortality than sarcopenia or osteoporosis alone [11, 12] However, the study by Scott et al. only included men, and the study by Balogun et al. had limited sample size to conclude whether osteosarcopenia increases the risk more than having sarcopenia or osteoporosis alone in the general population.

This study aimed to compare the risk of any fracture, major osteoporotic fracture, hip fracture and mortality among individuals with probable sarcopenia only, osteoporosis only, osteosarcopenia, and those with neither condition, using a nationwide cohort study representative of the general Finnish population aged 55 and older, with follow-up data spanning up to 19 years.

## Methods

### Study population

This study utilizes data from the Health 2000 survey, a comprehensive health examination survey conducted across Finland in 2000–2001. A random sample of 10,000 individuals aged 18 and above was drawn from the national population register, employing a stratified two-stage cluster sampling method. The survey included both community-dwelling and institutionalized individuals residing in mainland Finland. Detailed descriptions of the survey’s methodology have been published previously [13]. In total, 8,028 participants took part in the survey, with 3,439 of them being 55 years or older. For this study, we focused on participants aged 55 and above who had available data on sarcopenia and osteoporosis, resulting in a sample size of 2,506 (72.9%).

### Measurement of probable sarcopenia, osteoporosis and osteosarcopenia

Grip strength was assessed using an electronic device (Good Strength, IGS01, Metitur Oy, Finland) with participants seated and their elbows resting on a table while holding the device’s handle [13, 14]. The measurement was conducted twice, with a 30-second interval between attempts. If the difference between the two measurements exceeded 10%, a third measurement was taken. The highest value recorded was used for analysis. Grip strength measurement devices were calibrated on regular intervals using standard weights. Probable sarcopenia was identified based on the EWGSOP2 criteria, with low grip strength defined as less than 27 kg for men and less than 16 kg for women [4]. 

Bone mineral density was evaluated using a calcaneal ultrasound device (Sahara Clinical Bone Sonometer, Hologic, Waltham, Massachusetts). The devices were tested using a phantom object provided by the manufacturer before each measurement. The Quantitative Ultrasound Index (QUI), provided by the manufacturer, served as the indicator of bone mineral density. QUI was calculated from the speed of sound (SOS) and broadband ultrasound attenuation (BUA) using formula:

$$\:QUI\:=\:0.41\:\times\:\:SOS\:+\:0.41\:\times\:\:BUA\:-\:571$$

Osteoporosis was defined as a bone density measurement with a T-score less than − 2.5, based on ultrasound results. The reference group for the T-score consisted of 30–35-year-old women without chronic illness or disability (*n* = 300) [1]. Additionally, participants reporting a prior diagnosis of osteoporosis confirmed by DXA (dual-energy X-ray absorptiometry) were classified as having osteoporosis, regardless of their ultrasound-based bone density measurement. In total, 163 participants (6.5%) self-reported a prior osteoporosis diagnosis.

Osteosarcopenia was defined as having both probable sarcopenia and osteoporosis. Study participants were assigned into four groups: no sarcopenia and no osteoporosis (‘reference group’ from hereon), probable sarcopenia only, osteoporosis only, and osteosarcopenia.

### Assessment of bone fractures

The follow-up information about bone fractures was obtained from the National Hospital Discharge Register by using the national identification numbers assigned to each Finnish resident.

Fractures were identified by the ICD-10 codes corresponding to the care event and three different fracture outcomes were defined: any fracture, major osteoporotic fractures and hip fractures. The following ICD-10 codes and all their sub-codes were included in the ‘any fracture’ outcome: S02, S12, S22, S32, S42, S52, S62, S72, S82, and S92. Major osteoporotic fractures, as defined by the WHO, included hip (S72), clinical spine (S12, S22, S32), shoulder (S42), and wrist fractures (S52). To comply with the WHO’s definition of Major osteoporotic fracture, exclusions were made for non-vertebral thoracic and pelvic fractures (S22.2-S22.9, S32.1-S32.8), non-wrist and non-shoulder upper extremity fractures (S52.0-S52.4, S52.7-S52.9) and diaphyseal and distal femur fractures (S72.3, S72.4, and S72.7-S72.9) [15]. Hip fractures included events coded as S72.0, S72.1 and S72.2. Because high-energy impacts may cause fractures regardless of bone health or sarcopenia status, we excluded all fractures resulting from a high-energy impact (traffic accidents, falls down stairs or ladders, injuries from motorized machines).

To exclude participants with earlier bone fractures for sensitivity analysis purposes, information on fractures preceding the enrollment for the study was also gathered from the National Hospital Discharge Register. In total, the data on fractures spanned from November 21 st, 1994, to December 31 st, 2019.

The follow-up for each fracture outcome (any fracture, major osteoporotic fracture, and hip fracture) continued until the occurrence of the specific fracture type being analyzed, the date of death, or the end of the study period (December 31 st, 2019), whichever came first. Participants experiencing a different fracture type remained in the study for the outcome of interest.

### Mortality ascertainment

Mortality was followed until the date of death or end of follow-up, i.e. 31 st December 2019. The Health 2000 dataset was linked to Statistics Finland’s Causes of Death Register, which includes information on the date and cause of death, by utilizing the personal identity codes assigned to each Finnish resident.

### Demographic and lifestyle-related covariates

Information on age and sex were obtained from the population register. Education, smoking, physical activity and mobility limitations were obtained from survey questionnaires. Education was categorized as basic, secondary or higher. Smoking was categorized dichotomously as current and ex-smokers, or never smokers. Physical activity was categorized either exercise training (“Leisure time includes strenuous physical exercise at least 3 hours per week”), active (“Leisure time includes walking, bicycling and other forms of physical activity at least 4 hours per week”) or inactive. (“Leisure time consists of reading, television, or activities not involving physical activity”) [16] To measure mobility, subjects were asked “Are you able to walk about half a kilometer without resting?” and “Are you able to climb one flight of stairs without resting?”. Any difficulty in either task indicated a mobility limitation.

### Statistical analysis

Comparisons between osteosarcopenia groups at the baseline were performed using Student’s t test for continuous variables and chi-square test for categorical variables.

To examine the association between osteosarcopenia groups and fracture risk and mortality, we used three different analytic approaches. First, we conducted unadjusted survival analyses using Kaplan-Meier estimates with 95% confidence intervals (PROC LIFETEST in SAS 9.4). Second, we performed multivariable survival analyses using the Cox proportional hazards model (PROC PHREG in SAS 9.4) to estimate adjusted hazard ratios (HR) with 95% confidence intervals (CI). Initially we adjusted the analyses for age and sex (Model 1). In Model 2 we additionally adjusted for education, smoking, and physical activity; and in Model 3 we additionally adjusted for mobility limitation. Due to the relatively high proportion of missing physical activity data (up to 15.6%; see Table 1), we opted to retain subjects even if their physical activity data were missing. For all other covariates, participants with missing data were excluded. Finally, in Model 4, to further account for death as a competing risk for fractures, we utilized the Fine-Gray method incorporating covariates from Model 3 [17]. We tested the proportional hazards assumption using Schoenfeld residuals.

We also conducted several sensitivity analysis to test robustness of our findings. First, to eliminate the risk of previous fractures being identified as new incidents, we excluded all subjects with previous fracture of the respective type (e.g. excluded all those with a previous hip fracture from the hip fracture hazard analysis). Second, we included only fractures resulting from low-energy impacts (falls on the same level, falls from bed, falls due to ice and snow) to capture only fractures related to fragility. Finally, we limited the follow-up to December 31 st, 2010, i.e. about 10 years, to minimize the bias related to changing health status and especially changes in the exposure variables. For all sensitivity analyses we used the Fine-Gray method for fractures, and Cox Proportional Hazards analysis for mortality, both with adjustment Model 3.

All analyses were conducted using SAS software version 9.4 (SAS Institute Inc., Cary, North Carolina, United States). The analysis code used for this study is available in the supplementary materials.

## Results

 The current study sample was slightly younger than the whole Health 2000 study sample in those aged 55 and older. A higher proportion of participants were engaged in physical activity and had highest education level. Fewer participants in the study sample had mobility limitations compared to the whole Health 2000 study participants. Those who did not have muscle strength or bone density measurements available also had more missing data on the selected covariates. (Table S1)

The mean age at baseline varied across groups, with the reference group having the lowest (66.0 years, SD 8.2) and the osteosarcopenia group having the highest mean age (80.4 years, SD 8.6). Large majority of the participants in the osteoporosis only and osteosarcopenia groups were women (90% and 88%). Detailed baseline characteristics across osteosarcopenia groups are shown in Table 1.

**Table 1:** Baseline characteristics of the osteosarcopenia groups

	Reference group	Probable sarcopenia only	Osteoporosis only	Osteosarcopenia	*p*-value
Number of subjects	1966	234	184	122	
Age (years), mean (SD)	66.0 (8.2)	75.2 (9.5)	72.5 (9.1)	80.4 (8.6)	< 0.001
Female, n (%)	1052 (53.5)	147 (62.8)	166 (90.2)	107 (87.7)	< 0.001
Education					
Higher, n (%)	318 (16.2)	24 (10.3)	20 (10.9)	12 (9.9)	< 0.001
Secondary, n (%)	430 (21.9)	36 (15.5)	48 (26.1)	19 (15.7)	
Basic, n (%)	1211 (61.6)	173 (74.3)	116 (63.0)	90 (74.4)	
Missing, n (%)	7 (< 1)	1 (< 1)	0 (0)	1 (< 1)	
Current or past smoking, n (%)	818 (41.6)	93 (39.7)	43 (23.4)	27 (22.1)	< 0.001
Missing, n (%)	8 (< 1)	1 (< 1)	1 (< 1)	2 (1.6)	
Physical activity					
Exercise training, n (%)	273 (13.9)	11 (4.7)	18 (9.8)	3 (2.5)	< 0.001
Active, n (%)	1185 (60.3)	92 (39.3)	93 (50.5)	28 (23.0)	
Inactive, n (%)	459 (23.4)	106 (45.3)	64 (34.8)	72 (59.0)	
Missing, n (%)	49 (2.5)	25 (10.7)	9 (4.9)	19 (15.6)	
Mobility limitation, n (%)	359 (18.3)	129 (55.1)	77 (41.9)	96 (78.7)	< 0.001
Missing, n (%)	11 (< 1)	3 (1.3)	3 (1.6)	4 (3.3)	

**Fig. 1:**
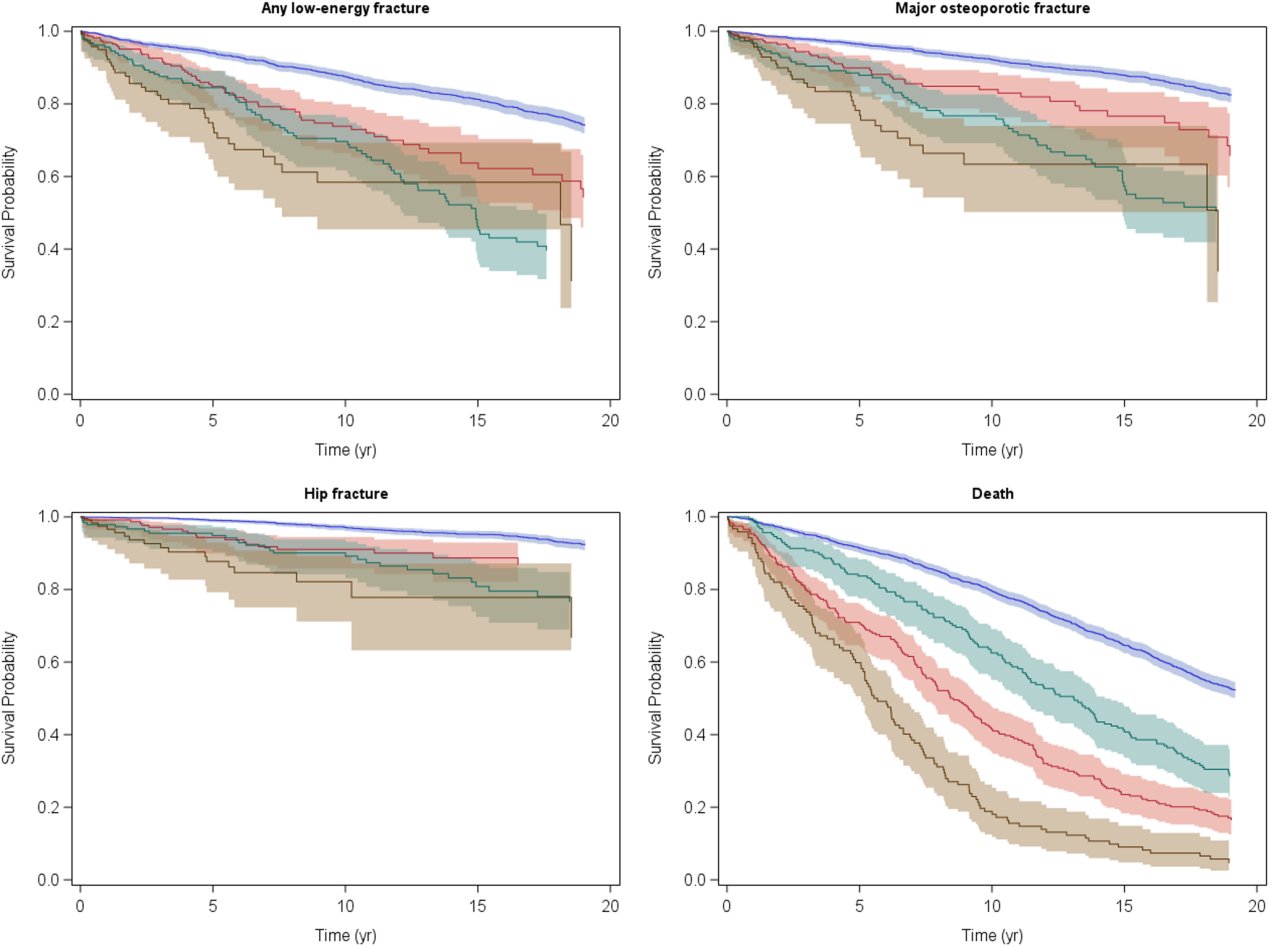
Kaplan-Meier survival curves with 95% confidence intervals for any low-energy fracture, major osteoporotic fracture, hip fracture and death. Blue: reference group. Red: probable sarcopenia only group. Green: osteoporosis only group. Brown: osteosarcopenia group

During the follow-up 580 participants (23.1%) sustained a fracture of any type, 403 (16.1%) a major osteoporotic fracture, 176 (7.0%) a hip fracture and 1,375 (54.9%) died. Figure 1 presents Kaplan-Meier survival curves for each outcome separately.

### Any low-energy fracture

The mean follow-up time to any low-energy fracture occurrence, death or end of follow-up was 12.5 years. The hazard of any low-energy fracture was higher in probable sarcopenia only (HR 1.35, 95% CI 1.01–1.81), osteoporosis only (HR 1.97, 95% CI 1.52–2.56), and osteosarcopenia groups (HR 1.74, 95% CI 1.18–2.57) compared to the reference group even after adjusting for demographics, lifestyle factors and mobility limitation (Table 2). When competing risk of death was accounted for, only the osteoporosis only group had a significantly higher hazard (HR 1.86, 95% CI 1.42–2.43) compared to the reference group. When comparing to the osteosarcopenia group, osteoporosis only group showed a borderline statistically significantly higher hazard of any low-energy fracture (HR 1.57, 95% CI 1.00–2.47), while probable sarcopenia only and reference groups showed no difference (Table 3).

### Major osteoporotic fracture

The mean follow-up period for major osteoporotic fracture outcome was 11.6 years. The hazard for major osteoporotic fracture was significantly higher in osteoporosis only and osteosarcopenia groups but not in probable sarcopenia only group compared to the reference group in age and sex adjusted model, as well as when additionally adjusted for demographic factors, lifestyle factors and mobility limitation (Table 2). When competing risk of death was accounted for, only the osteoporosis only group had a significantly higher hazard compared to the reference group (HR 1.85, 95% CI 1.35–2.54). The hazard of major osteoporotic fracture was not different for osteosarcopenia group when compared to the probable sarcopenia only and osteoporosis only groups (Table 3).

### Hip fracture

The mean follow-up period for hip fracture outcome was 12.1 years. The osteoporosis only and the osteosarcopenia groups had a higher hazard of hip fracture in age and sex adjusted model as well as in the model additionally adjusting for demographic factors, lifestyle factors and mobility limitation, whereas the probable sarcopenia only group showed no difference to the reference group. (Table 2). This remained statistically significant only for the osteoporosis only group when accounting for the competing risk of death (HR 1.84, 95%CI 1.13–2.99). There were no differences between the osteosarcopenia group and the probable sarcopenia only or the osteoporosis only groups (Table 3).

### Death

The mean follow-up period for mortality was 12.3 years. Mortality was significantly higher in the probable sarcopenia only (HR 1.45, 95% CI 1.20–1.75) and osteosarcopenia groups (HR 1.84, 95% CI 1.45–2.32) but not in the osteoporosis only group (HR 1.22, 95% CI 0.98–1.51) compared to the reference group after adjusting for demographics, lifestyle factors and mobility limitation (Table 2). When compared to the osteosarcopenia group, the osteoporosis only group had significantly lower mortality (HR 0.66, 95% CI 0.50–0.89) while mortality in the probable sarcopenia only group did not significantly differ from mortality in the osteosarcopenia group (HR 0.79, 95% CI 0.60–1.03) (Table 3).

### Sensitivity analyses

 The main analyses were repeated including only subjects with no previous fracture of the respective type, including only low energy fractures, and restricting the follow-up period to approximately 10 years. When subjects with previous fractures of the same type were excluded, the osteoporosis only group was still the only group with higher hazard of any low-energy fracture, major osteoporotic fracture or hip fracture compared to the reference group in the fully adjusted model (Model 4), as was the case in the main results. (Table S2) Hip fracture hazard was no longer statistically significantly higher in the osteoporosis only group compared to the reference group group when restricting the analysis to fractures with a confirmed low-energy cause, rather than excluding only those with a known high-energy cause (HR 1.65, 95% CI 0.99–2.76)(Table S3). Similarly, when restricting the follow-up period to 10 years, the results were virtually unchanged compared to the main results, with the exception of hip fracture hazard in the osteoporosis only group barely not being statistically significantly different compared to the reference group group (HR 1.94, 95%CI 0.99–3.79). (Table S4)

**Table 2:** Hazard ratios of any fracture, major osteoporotic fracture, hip fracture and death across osteosarcopenia groups

				Model ^1^	Model^2^	Model^3^	Model^4^
		n	Number of events (%^5^)	HR (95% CI)	HR (95% CI)	HR (95% CI)	HR (95% CI)
Any low-energy fracture	Reference group	1966	405 (20.6)	1.00 (Reference)	1.00 (Reference)	1.00 (Reference)	1.0 (Reference)
	Probable sarcopenia only	234	60 (25.6)	**1.44 (1.09–1.91)**	**1.40 (1.06–1.86)**	**1.35 (1.01–1.81)**	1.11 (0.82–1.51)
	Osteoporosis only	184	79 (42.9)	**2.03 (1.57–2.62)**	**2.02 (1.56–2.61)**	**1.97 (1.52–2.56)**	**1.86 (1.42–2.43)**
	Osteosarcopenia	122	36 (29.5)	**1.88 (1.29–2.72)**	**1.84 (1.26–2.67)**	**1.74 (1.18–2.57)**	1.18 (0.76–1.82)
Major osteoporotic fracture	Reference group	1966	269 (13.7)	1.00 (Reference)	1.00 (Reference)	1.00 (Reference)	1.0 (Reference)
	Probable sarcopenia only	234	40 (17.1)	1.30 (0.92–1.83)	1.25 (0.88–1.78)	1.19 (0.83–1.70)	0.96 (0.66–1.39)
	Osteoporosis only	184	63 (34.2)	**2.04 (1.53–2.74)**	**2.03 (1.51–2.72)**	**1.96 (1.46–2.65)**	**1.85 (1.35–2.54)**
	Osteosarcopenia	122	31 (25.4)	**1.97 (1.30–2.98)**	**1.95 (1.28–2.95)**	**1.82 (1.18–2.80)**	1.20 (0.74–1.93)
Hip fracture	Reference group	1966	112 (5.7)	1.00 (Reference)	1.00 (Reference)	1.00 (Reference)	1.0 (Reference)
	Probable sarcopenia only	234	19 (8.1)	1.28 (0.77–2.13)	1.27 (0.76–2.12)	1.17 (0.68–2.01)	0.88 (0.50–1.54)
	Osteoporosis only	184	28 (15.2)	**1.96 (1.25–3.05)**	**1.96 (1.26–3.06)**	**2.02 (1.29–3.12)**	**1.84 (1.13–2.99)**
	Osteosarcopenia	122	17 (13.9)	**2.07 (1.17–3.66)**	**2.09 (1.18–3.73)**	**1.92 (1.04–3.55)**	1.19 (0.61–2.31)
Death	Reference group	1966	933 (47.5)	1.00 (Reference)	1.00 (Reference)	1.00 (Reference)	n/a
	Probable sarcopenia only	234	195 (83.3)	**1.77 (1.50–2.08)**	**1.61 (1.34–1.93)**	**1.45 (1.20–1.75)**	n/a
	Osteoporosis only	184	131 (71.2)	**1.36 (1.12–1.65)**	**1.30 (1.06–1.60)**	1.22 (0.98–1.51)	n/a
	Osteosarcopenia	122	116 (95.1)	**2.25 (1.82–2.80)**	**2.12 (1.69–2.67)**	**1.84 (1.45–2.32)**	n/a

^1^Model 1: adjusted for age and sex.

^2^ Model 2: adjusted for age, sex, smoking, education, and physical activity.

^3^Model 3: adjusted for age, sex, smoking, education, physical activity and mobility limitation.

^4^Model 4: Fine-Gray method adjusted for age, sex, smoking, education, physical activity and mobility limitation.

^5^Percentage of subjects within the respective group who experienced the event of interest during follow-up

**Table 3:** Osteosarcopenia group used as 1.0 (Reference). Hazard ratios of any fracture, major osteoporotic fracture, hip fracture and death across osteosarcopenia groups

				Model 1^1^	Model 2^2^	Model 3^3^	Model 4^4^
		n	Number of events (%^5^)	HR (95% CI)	HR (95% CI)	HR (95% CI)	HR (95% CI)
Any low-energy fracture	Osteosarcopenia	122	36 (29.5)	1.0 (Reference)	1.0 (Reference)	1.0 (Reference)	1.0 (Reference)
	Reference group	1966	405 (20.6)	**0.53 (0.37–0.77)**	**0.55 (0.37–0.79)**	**0.58 (0.39–0.85)**	0.85 (0.55–1.31)
	Probable sarcopenia only	234	60 (25.6)	0.77 (0.51–1.71)	0.76 (0.50–1.16)	0.78 (0.51–1.20)	0.94 (0.59–1.51)
	Osteoporosis only	184	79 (42.9)	1.08 (0.72–1.62)	1.10 (0.73–1.64)	1.13 (0.75–1.72)	**1.57 (1.00–2.47)**
Major osteoporotic fracture	Osteosarcopenia	122	31 (25.4)	1.0 (Reference)	1.0 (Reference)	1.0 (Reference)	1.0 (Reference)
	Reference group	1966	269 (13.7)	**0.51 (0.34–0.77)**	**0.51 (0.34–0.78)**	**0.52 (0.33–0.82)**	0.84 (0.52–1.35)
	Probable sarcopenia only	234	40 (17.1)	0.66 (0.41–1.06)	0.64 (0.40–1.04)	0.63 (0.37–1.05)	0.80 (0.47–1.35)
	Osteoporosis only	184	63 (34.2)	1.04 (0.67–1.61)	1.04 (0.67–1.62)	1.05 (0.65–1.69)	1.55 (0.95–2.53)
Hip fracture	Osteosarcopenia	122	17 (13.9)	1.0 (Reference)	1.0 (Reference)	1.0 (Reference)	1.0 (Reference)
	Reference group	1966	112 (5.7)	**0.48 (0.27–0.86)**	**0.48 (0.27–0.85)**	**0.55 (0.36–0.85)**	0.84 (0.43–1.64)
	Probable sarcopenia only	234	19 (8.1)	0.62 (0.32–1.21)	0.61 (0.31–1.18)	0.65 (0.40–1.07)	0.74 (0.35–1.56)
	Osteoporosis only	184	28 (15.2)	0.95 (0.51–1.75)	0.94 (0.50–1.74)	1.08 (0.69–1.71)	1.55 (0.79–3.04)
Death	Osteosarcopenia	122	116 (95.1)	1.0 (Reference)	1.0 (Reference)	1.0 (Reference)	n/a
	Reference group	1966	933 (47.5)	**0.44 (0.36–0.55)**	**0.47 (0.37–0.59)**	**0.55 (0.43–0.69)**	n/a
	Probable sarcopenia only	234	234 (100)	**0.79 (0.62–0.99)**	**0.76 (0.59–0.98)**	0.79 (0.60–1.03)	n/a
	Osteoporosis only	184	184 (100)	**0.60 (0.47–0.78)**	**0.61 (0.47–0.81)**	**0.66 (0.50–0.89)**	n/a

^1^Model 1: adjusted for age and sex

^2^ Model 2: adjusted for age, sex, smoking, level of education, and physical activity

^3^ Model 3: adjusted for age, sex, smoking, level of education, physical activity and mobility limitation

^4^ Model 4: Fine-Gray method adjusted for age, sex, smoking, level of education, physical activity and mobility limitation

^5^ Percentage of subjects within the respective group who experienced the event of interest during follow-up

## Discussion

This population-based study investigated the association of probable sarcopenia, osteoporosis, and osteosarcopenia with the risk of low-energy fractures and mortality. All three conditions were associated with an increased hazard of any low-energy fractures, major osteoporotic fractures, hip fractures and death compared to having neither sarcopenia nor osteoporosis. When comparing to the osteoporosis only and sarcopenia only groups, the osteosarcopenic group did not show an increased fracture risk. However, the osteoporosis only group had lower mortality compared to the osteosarcopenia group. Thus, while both probable sarcopenia and osteoporosis are risk factors for fractures and mortality, sarcopenia seems to have a stronger association with mortality, while osteoporosis has a stronger association with fracture risk.

Consistent with prior studies [3, 7, 10–12, 18], we found both probable sarcopenia and osteoporosis to be associated with an increased hazard of fractures and mortality (depending on the used statistical model and duration of follow-up). When death was accounted for as a competing risk for fractures, only the osteoporosis only group had an increased hazard of any fracture, major osteoporotic fracture, and hip fracture compared to the reference group.

This study aimed to elucidate whether osteosarcopenia poses greater risk for fractures and mortality compared to sarcopenia or osteoporosis alone. Our findings corroborate with previous population-based studies from Tasmania by Balogun et al. (2019) and from Australia by Scott et al. (2019) [11, 12]. The main difference between our results and the results of Balogun et al. is that they found no difference in mortality between those with low grip strength compared to those with normal grip strength in their multivariable adjusted analysis [11]. The lack of association between low grip strength and mortality in their findings may be due to the differences in defining low grip strength, chosen covariates in their statistical tests or underpowered sample size. Similarly, Scott et al. found that those with osteosarcopenia had a similar fracture risk as those with osteopenia/osteoporosis alone [12]. 

Taking the results from Scott et al., Balogun et al., and our study together, identifying individuals with osteosarcopenia has not been shown to be more effective in predicting fracture risk than identifying those with either osteoporosis or low grip strength alone. However, since both grip strength and BMD are continuously and inversely associated with fracture risk, it is important to measure grip strength in addition to BMD when assessing overall fracture risk. Indeed, it has been suggested that assessing sarcopenia brings additional value in predicting fracture risk beyond the current version of the FRAX^®^ tool [6, 19, 20]. While no clinical trials have been conducted to conclude the effectiveness of targeted treatment of sarcopenia on reducing falls or fractures, exercise in general substantially reduces falls in those aged 60 and older [21]. It is plausible that those with sarcopenia gain even more benefit from exercise in terms of reducing the risk of falls and subsequent fractures. In summary, assessing both bone and muscle health is crucial in fracture risk evaluation. Future studies should determine how to best integrate these factors into clinical practice, whether by refining the FRAX^®^ tool or through alternative approaches.

This study benefits from a large nationwide sample that is representative of the Finnish general population. The National Hospital Discharge Register is reliable and has nationwide coverage. The coverage and quality of the Causes of Death Register kept by Statistics Finland is excellent. The duration of follow-up was exceptionally long, and the number of events of interest was adequate for a well-powered analysis. The methods for assessing bone mineral density and grip strength are well-established.

Nevertheless, some limitations should be recognized. Our total sample allowed us to detect meaningful differences between the probable sarcopenia, osteoporosis-only, and osteosarcopenia groups versus reference group. However, the osteosarcopenia group itself was small, and results comparing it with other groups should be viewed cautiously. The National Hospital Discharge Register is not fully complete, as fractures that do not require hospitalization may be treated outside hospitals (e.g. local healthcare centers and private clinics). The true number of fractures may thus be higher than presented here. Additionally, while calcaneal ultrasound-based bone densitometry has been shown to predict fracture risk with similar performance to DXA, there are no agreed criteria for diagnosing osteoporosis using calcaneal ultrasound [22, 23]. Lastly, this study lacks appendicular muscle mass assessment, and therefore diagnosis of sarcopenia could not be considered confirmed, but rather probable in those with low grip strength [4]. Consequently, the findings need to be interpreted with caution and further studies on the topic are warranted.

In conclusion, both probable sarcopenia and osteoporosis, as well as osteosarcopenia, were found to increase the risk of low-energy fractures and mortality. However, osteosarcopenia did not appear to pose an additive risk for fractures. On the other hand, osteosarcopenia was found to associate with higher mortality compared to osteoporosis only highlighting the importance of identifying sarcopenia among older adults. Further research is necessary to determine the best ways to incorporate sarcopenia assessment into comprehensive fracture risk evaluation.

## Supplementary Information

Below is the link to the electronic supplementary material.

Supplementary Material 1

Supplementary Material 2

## Data Availability

No datasets were generated or analysed during the current study.
