# Supplementary material for: Osteosarcopenia as a risk factor for fractures and mortality – 19-year follow-up of a population-based sample
Source: Aging Clin Exp Res. 2025 Nov 6;37(1):319. doi: 10.1007/s40520-025-03229-8 (PMC12592282; doi:10.1007/s40520-025-03229-8)
Supplement: Supplementary file 1 — Supplementary Material 1 [file 40520_2025_3229_MOESM1_ESM.pdf]

Table S1: Characteristics of study population and those who were excluded from the study population

	Study population, n=2506	Excluded from the study population, n=933	p-value
Age (SD)	68.0 (9.3)	75.4 (11.0)	<0.01
Female, n (%)	1472 (58.7)	609 (65.3)	<0.01
Smoking, n (%)			
Yes	981 (39.2)	189 (20.3)	<0.01
No	1513 (60.4)	342 (36.7)	
Missing	12 (0.5)	402 (43.1)	
Education, n (%)			
Higher	374 (14.9)	57 (6.1)	<0.01
Secondary	533 (21.3)	110 (11.8)	
Basic	1590 (63.5)	493 (52.8)	
Missing	9 (0.4)	273 (29.3)	
Physical activity, n (%)			
Sedentary	701 (28.0)	217 (23.3)	<0.01
Active	1398 (55.8)	81 (8.7)	
Exercise training	305 (12.2)	25 (2.7)	
Missing	102 (4.1)	610 (65.4)	
Mobility limitation, n (%)			
Yes	661 (26.4)	421 (45.1)	<0.01
No	1824 (72.8)	248 (26.6)	
Missing	21 (0.8)	264 (28.3)	

Table S2: All subjects with previous fracture of the respective type excluded (n=2354 for Any fracture; n=2405 for Major osteoporotic fracture; n=2458 for Hip fracture). Hazard ratios of any fracture, major osteoporotic fracture, and hip fracture across osteosarcopenia groups.

		n	Number of events (%[1])	HR (95% CI)
Any low-energy fracture	No sarcopenia, no osteoporosis	1884	376 (20.0)	1.0 (Reference)
	Probable sarcopenia only	213	51 (23.9)	1.12 (0.81 – 1.55)
	Osteoporosis only	166	66 (39.8)	**1.75 (1.31 – 2.34)**
	Osteosarcopenia	91	27 (29.7)	1.28 (0.79 – 2.07)
Major osteoporotic fracture	No sarcopenia, no osteoporosis	1919	255 (13.3)	1.0 (Reference)
	Probable sarcopenia only	218	35 (16.1)	0.96 (0.65 – 1.42)
	Osteoporosis only	171	56 (32.8)	**1.82 (1.31 – 2.53)**
	Osteosarcopenia	97	25 (25.8)	1.28 (0.77 – 2.12)
Hip fracture	No sarcopenia, no osteoporosis	1946	110 (5.7)	1.0 (Reference)
	Probable sarcopenia only	227	16 (7.1)	0.82 (0.47 – 1.45)
	Osteoporosis only	179	27 (15.1)	**1.74 (1.07 – 2.85)**
	Osteosarcopenia	106	13 (12.3)	1.10 (0.55 – 2.19)

Fine-Gray method adjusted for age, sex, smoking, education, physical activity and mobility limitation.
[1]Percentage of subjects within the respective group who experienced the event of interest during follow-up

Table S3: Only low-energy fractures included (n=2481), as opposed to excluding high-energy fractures. Hazard ratios of any fracture, major osteoporotic fracture, and hip fracture across osteosarcopenia groups.

		n	Number of events (%[1])	HR (95% CI)
Any low-energy fracture	No sarcopenia, no osteoporosis	1953	292 (15.0)	1.0 (Reference)
	Probable sarcopenia only	230	46 (20.0)	1.10 (0.78 – 1.54)
	Osteoporosis only	181	67 (37.0)	**2.01 (1.49 – 2.71)**
	Osteosarcopenia	117	30 (25.6)	1.20 (0.75 – 1.91)
Major osteoporotic fracture	No sarcopenia, no osteoporosis	1953	207 (10.7)	1.0 (Reference)
	Probable sarcopenia only	230	30 (13.0)	0.92 (0.60 – 1.40)
	Osteoporosis only	181	52 (28.7)	**1.92 (1.35 – 2.74)**
	Osteosarcopenia	117	26 (22.2)	1.26 (0.75 – 2.11)
Hip fracture	No sarcopenia, no osteoporosis	1953	103 (5.3)	1.0 (Reference)
	Probable sarcopenia only	230	14 (6.1)	0.74 (0.41 – 1.33)
	Osteoporosis only	181	25 (13.8)	1.65 (0.99 – 2.76)
	Osteosarcopenia	117	15 (12.8)	1.18 (0.60 – 2.31)

Fine-Gray method adjusted for age, sex, smoking, education, physical activity and mobility limitation.
[1]Percentage of subjects within the respective group who experienced the event of interest during follow-up

Table S4: Follow-up period restricted to December 31st, 2010 (n=2481). Hazard ratios of any low-energy fracture, major osteoporotic fracture, hip fracture and death across osteosarcopenia groups.

		n	Number of events (%[1])	HR (95% CI)
Any low-energy fracture	No sarcopenia, no osteoporosis	1953	227 (13.9)	1.0 (Reference)
	Probable sarcopenia only	230	45 (19.6)	1.26 (0.89 – 1.79)
	Osteoporosis only	181	48 (26.5)	**1.76 (1.25 – 2.48)**
	Osteosarcopenia	117	32 (27.4)	1.48 (0.93 – 2.35)
Major osteoporotic fracture	No sarcopenia, no osteoporosis	1953	141 (7.2)	1.0 (Reference)
	Probable sarcopenia only	230	28 (12.2)	1.10 (0.70 – 1.73)
	Osteoporosis only	181	36 (19.9)	**1.77 (1.17 – 2.68)**
	Osteosarcopenia	117	27 (23.1)	1.56 (0.92 – 2.63)
Hip fracture	No sarcopenia, no osteoporosis	1953	51 (2.6)	1.0 (Reference)
	Probable sarcopenia only	230	14 (6.1)	1.24 (0.64 – 2.42)
	Osteoporosis only	181	16 (8.8)	1.94 (0.99 – 3.79)
	Osteosarcopenia	117	14 (12.0)	1.69 (0.79 – 3.64)
Death	No sarcopenia, no osteoporosis	1953	404 (20.7)	1.0 (Reference)
	Probable sarcopenia only	230	132 (57.4)	**1.39 (1.11 – 1.75)**
	Osteoporosis only	181	68 (37.6)	1.24 (0.94 – 1.64)
	Osteosarcopenia	117	96 (82.1)	**1.97 (1.51 – 2.56)**

Fractures: Fine-Gray method adjusted for age, sex, smoking, education, physical activity and mobility limitation. Death: Cox Proportional Hazards method adjusted for age, sex, smoking, education, physical activity and mobility limitation. [1]Percentage of subjects within the respective group who experienced the event of interest during follow-up
